# Losac and Lopap Recombinant Proteins from *Lonomia obliqua* Bristles Positively Modulate the Myoblast Proliferation Process

**DOI:** 10.3389/fmolb.2022.904737

**Published:** 2022-06-29

**Authors:** Angela María Alvarez, Miryam Paola Alvarez-Flores, Carlos DeOcesano-Pereira, Mauricio Barbugiani Goldfeder, Ana Marisa Chudzinski-Tavassi, Vanessa Moreira, Catarina Teixeira

**Affiliations:** ^1^ Centre of Excellence in New Target Discovery -CENTD-, Butantan Institute, São Paulo, Brazil; ^2^ Pharmacology Department, Escola Paulista de Medicina, Universidade Federal de São Paulo, São Paulo, Brazil; ^3^ Innovation and Development Labororatory, Butantan Institute, São Paulo, Brazil; ^4^ Pharmacology Laboratory, Butantan Institute, São Paulo, Brazil

**Keywords:** myoblast, proliferation, differentiation, prostaglandin E_2_, inflammation

## Abstract

The pursuit of better therapies for disorders creating deficiencies in skeletal muscle regeneration is in progress, and several biotoxins are used in skeletal muscle research. Since recombinant proteins derived from Lonomia obliqua bristles, recombinant Lonomia obliqua Stuart-factor activator (rLosac) and recombinant Lonomia obliqua prothrombin activator protease (rLopap) act as cytoprotective agents and promote cell survival, we hypothesize that both rLosac and rLopap favour the skeletal muscle regeneration process. In the present work, we investigate the ability of these recombinant proteins rLosac and rLopap to modulate the production of key mediators of the myogenic process. The expression of myogenic regulatory factors (MRFs), cell proliferation, the production of prostaglandin E2 (PGE_2_) and the protein expression of cyclooxygenases COX-1 and COX-2 were evaluated in C2C12 mouse myoblasts pre-treated with rLosac and rLopap. We found an increased proliferation of myoblasts, stimulated by both recombinant proteins. Moreover, these proteins modulated PGE_2_ release and MRFs activities. We also found an increased expression of the EP4 receptor in the proliferative phase of C2C12 cells, suggesting the involvement of this receptor in the effects of PGE_2_ in these cells. Moreover, the recombinant proteins inhibited the release of IL-6 and PGE_2_, which is induced by an inflammatory stimulus by IL-1β. This work reveals rLopap and rLosac as promising proteins to modulate processes involving tissue regeneration as occurs during skeletal muscle injury.

## Introduction

Skeletal muscle is the most abundant tissue in the human body, with a pivotal role in energy and protein metabolism and the generation of force for locomotion and stability. These essential functions can be disrupted by muscle injury from dystrophic diseases, cachexia of tumoral causes and advanced age. The loss of muscle mass or function due to injury may limit the activities of daily living and reduce quality of life. Damaged skeletal muscle has the intrinsic capacity to regenerate and repair itself through myogenesis. The myogenic response involves a complex and orchestrated sequence of events from the activation of stem muscle cells, called satellite cells, to fibre repair and mature tissue renewal. Several stimuli promote the differentiation of quiescent satellite cells into myoblasts, which in turn, proliferate, differentiate and fuse to form multinucleated muscle fibres to repair damaged myofibres or to form new ones (Schmidt et al., 2019; Forcina et al., 2020). These processes are finely regulated by transcription factors, such as Pax7 and Pax3, and by myogenic regulatory factors (MRFs) including MyoD, Myf5, Myf6, and myogenin, which are expressed and activated in a coordinated sequence (Ferri et al., 2009; Schmidt et al., 2019). Activation of the innate immune response also plays an important role during regeneration events, and both proliferation and differentiation phenomena are also regulated by secreted inflammatory mediators (Forcina et al., 2020; Howard et al., 2020). A shift from pro-to anti-inflammatory signalling within days of muscle injury decreases the local inflammatory response and drives the later phases of myogenesis (Arnold et al., 2007; Howard et al., 2020). The inflammatory mediators involved in the development of the regeneration process, such as pro-inflammatory cytokines (Philippou et al., 2012; Londhe and Guttridge, 2015; Forcina et al., 2020) and cyclooxygenase (COX)–derived prostaglandins (Bondesen et al., 2004; Mendias et al., 2004; Monda et al., 2009; Novak et al., 2009; Mo et al., 2012; Mo et al., 2015; Alvarez et al., 2020) are well characterized. However, several lines of evidence indicate that while inflammatory signalling mediates the myogenic response to muscle damage, high concentrations of inflammatory cytokines, such as TNF-alpha (TNF-α), IL-6, and IL-1 beta (IL-1β) can inhibit muscle regeneration and trigger muscle wasting (Howard et al., 2020). Therefore, failure to resolve persistent pro-inflammatory signalling after muscle injury may dysregulate muscle regeneration, muscle repair and the maintenance of tissue mass.

Diverse anti-inflammatory and disease-modifying drugs are available to treat deficient skeletal muscle regeneration conditions. However, the prolonged use of these drugs is associated with severe side effects and is a matter of concern. Therefore, the investigation of new therapeutic agents that are more effective and safer is urgently needed. Toxins isolated from animal secretions have been used to find new therapeutic agents or prototipes for design of drugs useful to treat several human diseases (Herzig et al., 2020). In this context, the whole venom of the *Lonomia obliqua* caterpillar’s bristles was shown to induce differential cell proliferation effects in human glioma cell lines (Heinen et al., 2014). Two procoagulant proteins were isolated from this venom: the *Lonomia obliqua* Stuart factor activator (Losac) and the *Lonomia obliqua* prothrombin activator protease (Lopap). Losac belongs to the hemolin family of proteins (Alvarez Flores et al., 2006; Alvarez-Flores et al., 2011), which are multifunctional proteins exclusively expressed by the insect order Lepidoptera. Some of these proteins are involved in the regeneration of wounded tissue (Bettencourt et al., 1997; Bettencourt et al., 1999). Losac induced proliferation and cytoprotection in nutrient-deprived endothelial cells (Alvarez Flores et al., 2006). Its recombinant form–rLosac–inhibited the apoptosis of human fibroblasts and promoted cell survival in mouse cortical neurons (Bosch et al., 2016; Alvarez-Flores et al., 2019). It also improved wound healing in an *in vivo* model of a skin wound by preserving the extracellular matrix organization, including collagen type I, fibronectin and laminin expression (Sato et al., 2016). Lopap, in turn, belongs to the lipocalin family of proteins. Lipocalins are known for their role as protective factors and their involvement in regeneration and tissue repair processes (Spreyer et al., 1990; Kim et al., 2005; Playford et al., 2006). Lopap induced the expression of adhesion molecules in endothelial cells and was involved in the cells’ survival (Chudzinski-Tavassi et al., 2001; Fritzen et al., 2005). Recombinant Lopap (rLopap) has shown procoagulant properties (Reis et al., 2006) and inhibited the apoptosis of neutrophils and endothelial cells, evidencing its cytoprotective properties (Waismam et al., 2009).

Since recombinant Losac promotes cell proliferation and both rLosac and rLopap act as cytoprotective agents, we hypothesize that both rLosac and rLopap may favour skeletal muscle regeneration processes. Based on this working hypothesis, we investigated the effects of these *Lonomia obliqua*–derived recombinant proteins on 1) the proliferation and production of regulatory factors, such as transcription factors and prostaglandin E_2_ (PGE_2_), in myoblast cells and 2) the release of PGE_2_ and IL-6 upon stimulus by an inflammatory cytokine at the proliferation stage in myoblast cells.

## Materials and Methods

### Recombinant Protein Production

In this study, the cloning, heterologous expression and characterization of recombinant Losac was used, as previously described by Alvarez-Flores et al. (Alvarez-Flores et al., 2011). The cloning, expression and characterization of a recombinant form of Lopap, rLopap, was described by Reis et al. (Reis et al., 2006), along with its properties as a procoagulant and prothrombin activator.

### C2C12 Culture and Recombinant Proteins Treatment

**FIGURE 1:**
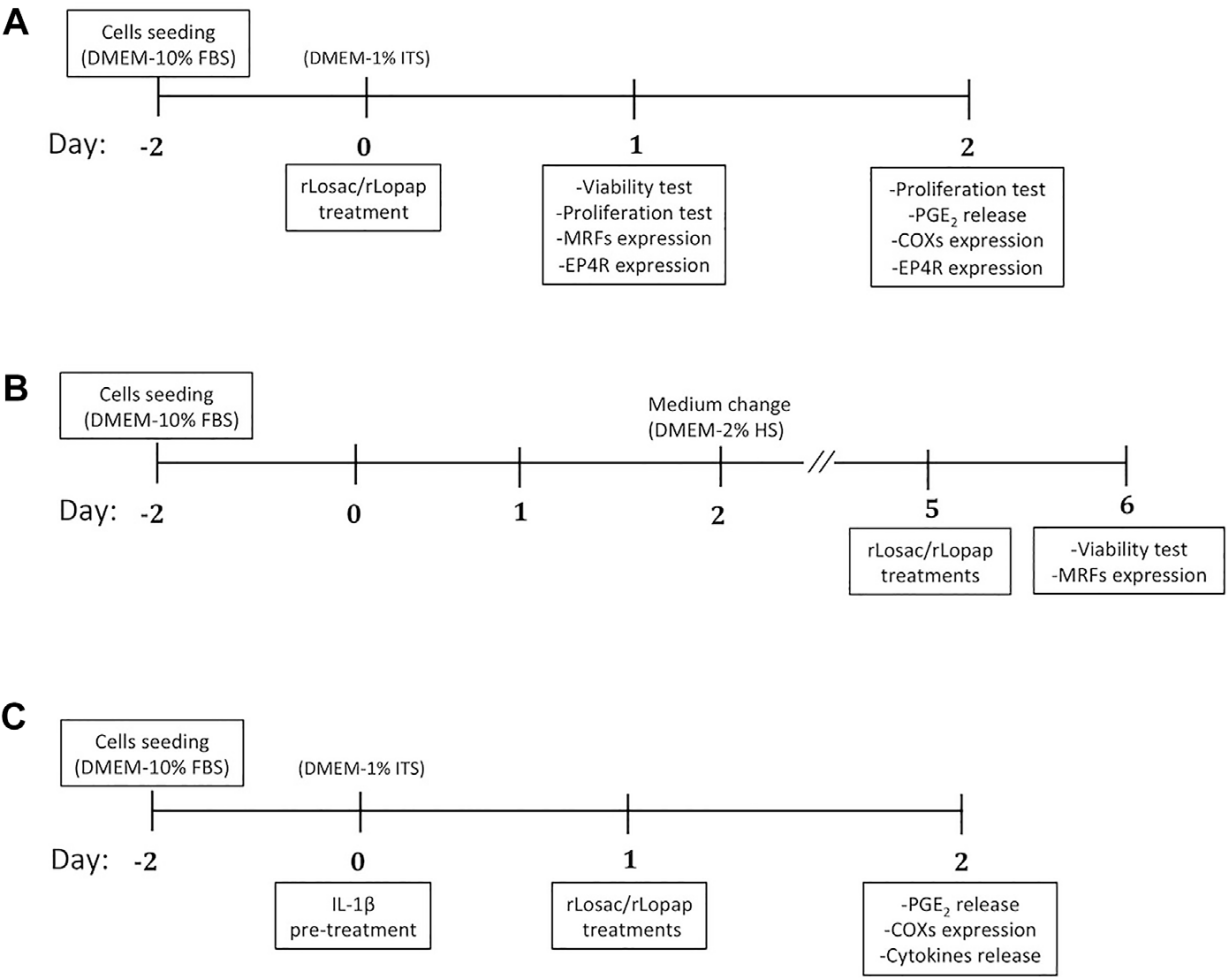
Workflows for studies performed under **(A)** proliferation, **(B)** differentiation, and **(C)** inflammatory/proliferation conditions. FBS: fetal bovine serum; HS: horse serum; ITS: insulin-transferrin-sodium.

The mouse myoblast cell line C2C12 (American Type Culture Collection, Manassas, United States) was used. These cells were grown in T75 flasks and left to 80% confluence in Dulbecco’s modified Eagle’s Medium (DMEM) (Gibco, Grand Island, NY, United States) supplemented with 10% heat-inactivated fetal bovine serum (FBS) (Gibco), 1% L-glutamine (Gibco) and 1% penicillin-streptomycin (Gibco) (DMEM-10% FBS), at 37°C in a 5% CO2 incubator. The studies were performed in two stages of the skeletal muscle cell regeneration (proliferation and differentiation) as well as under an inflammatory experimental condition, as follows (1): For studies in the proliferation stage, 0.1 × 10^4^ cells per well were seeded in 96 well plates (Corning, Corning, NY, United States) respectively, that had been previously coated with 2% gelatin (Sigma-Aldrich, Saint Louis, MO, United States), and maintained in culture for 48 h in DMEM-10% FBS to reach 60% confluence. Next, the medium was changed for the starving medium DMEM-1% insulin–transferrin–sodium (ITS) (Sigma-Aldrich), and cells were incubated either with 150 nM rLosac, or 150 nM rLopap, or DMEM-1% ITS only (control), for 24 or 48 h. At these time points, supernatants and cells were used for distinct analysis (Figure 1A). The concentration of rLosac and rLopap used in all experimental protocols was based on previous studies showing their cytoprotective effects (Fritzen et al., 2005; Alvarez Flores et al., 2006). (2) For studies in the differentiation stage, 0.1 × 10^4^ cells per well were seeded in 96-well plates, that had been previously coated with 2% gelatin and maintained in culture for 48 h in DMEM-10% FBS. After this time interval, the medium was changed for fresh DMEM-10% FBS and cells were maintained in culture for additional 48 h to reach 90% confluence. At this time point, cells were washed once with phosphate buffer saline (PBS), and then the medium was changed for DMEM supplemented with 2% horse serum (HS) (Sigma-Aldrich), for 72 h. Next, cells were incubated either with 150 nM rLosac, or 150 nM rLopap, or DMEM-2% HS only (control), for 24 h (Figure 1B). (3) To test the anti-inflammatory properties of recombinant proteins, assays were performed under an inflammatory condition using IL-1β as the inflammatory stimulus, on the basis of previous reports (Langen et al., 2001; Luo et al., 2003; Otis et al., 2014). The C2C12 cells were seeded at 2 × 10^4^ cells per well in six well-plates (Corning), that had been previously coated with 2% gelatin and maintained in culture for 48 h in DMEM-10% FBS to reach 60% confluence. At this time point, the medium was changed for starving medium DMEM-1% ITS, and cells were stimulated with the proinflammatory cytokine IL-1β (BioLegend, San Diego, CA, United States) at 10 ng/ml for 20 h. Next, 150 nM rLosac or 150 nM rLopap, or DMEM-1% ITS only (control) were added. 24 h after these treatments, supernatants and cells were used for distinct analysis (Figure 1C). The workflows for studies of the effects of rLosac and rLopap in proliferating and differentiating stages of C2C12 cells and in an inflammatory condition are demonstrated in Figure 1.

### Cell Proliferation by 5-Bromo-2′-Deoxyuridine Incorporation

To assess the cell proliferation, cells were seeded in 96-well plates following the protocol for the proliferative stage (Figure 1A). After the treatments with recombinant proteins, the BrdU incorporation was determined using the Cell Proliferation Assay Kit (Cell Signaling Technology, Danvers, MA, United States), following the manufacturer’s instructions. Four hours before the end of the treatments’ incubation, BrdU (1:1000) was added. Then, cells were fixed and denatured for 30 min. The plates were then washed and the primary antibody (1:250) was added for 1 h. The plates were washed again and incubated with the secondary antibody conjugated with horseradish peroxidase (HRP; 1:250) for 30 min. Finally, 3,3′,5,5′-tetramethylbenzidine substrate was added for 30 min, the stop solution was added and the plate was read at 450 nm in a spectrophotometer (SpectraMax, Molecular Devices, San Jose, CA, United States).

### Expression of Myogenic Regulatory Factors

For this assay C2C12 cells were seeded in black Advanced TC 96-well microplates (Greiner Bio-One, Kremsmünster, Austria). Cell culture and treatments with recombinant proteins were performed in both proliferation and differentiation stages according to the above-described protocols (Figures 1A,B). Immunofluorescence staining and image analysis were performed as described before (Alvarez et al., 2020). At 24 h after treatment with the recombinant proteins, cells were washed, fixed for 1 h with cold 4% paraformaldehyde, permeabilized for 5 min and blocked with 1% bovine serum albumin for 30 min. The primary antibodies rabbit anti-mouse Pax7, Myf5, MyoD (1:1000; Santa Cruz Biotechnology, Dallas, TX, United States), and myogenin (1:2000; Sigma-Aldrich) were added and incubated overnight at 4°C. After washing (three times), the cells were incubated with a Hoechst DNA-specific stain (1:3000; Thermo Fisher Scientific, Waltham, MA, United States) and anti-rabbit secondary antibodies conjugated to Alexa Fluor 488, 568, 647, and 594 respectively (1:1000; Thermo Fisher Scientific) at room temperature for 1 h. Finally, the plates were subjected to high content imaging analysis on MetaXpress High-Content Image Acquisition & Analysis Software (Molecular Devices). The system was used to acquire 16 images per well at 20× magnifications, and the quantitative data shown represent the mean fluorescence intensity of each protein, corrected with the secondary antibody control.

### Cyclooxygenases and EP4 Receptor Protein Expression by Western Blotting

C2C12 cells in six-well plates (Corning). Cell culture conditions and treatments with recombinant proteins were performed according to the protocol for the proliferation stage (Figure 1A) and also the protocol for inflammatory conditions (Figure 1C). At 24 or 48 h after treatments with the recombinant proteins, the medium was drained and cells were lysed with Laemmli buffer, and then an equal amount of protein was separated by 12% sodium dodecyl sulphate–polyacrylamide gel electrophoresis under reduced conditions. The separated proteins were transferred to nitrocellulose membranes (GE Healthcare, Chicago, IL, United States) using a wet blotter. The membranes were blocked with 5% non-fat powdered milk and incubated overnight at 4°C with primary antibody rabbit anti-mouse COX-1, COX-2 or EP4 (1:1000, 1:1500 and 1:250; Cayman Chemical, Ann Arbor, MI, United States). Then, the membranes were washed and incubated with a donkey anti-rabbit secondary antibody conjugated with HRP (1:2000; GE Healthcare). The conjugated peroxidase was detected by chemiluminescence with Immobilon Western (Millipore, Billerica, MA, United States), and the intensity of the bands was quantified by densitometry using ImageJ (NIH, Bethesda, MD, United States). The constitutive protein *β*-actin was used as the internal loading control, detected by anti-mouse anti-body (1:4000; Sigma-Aldrich) and an HRP-conjugated sheep anti-mouse secondary antibody (1:6000; GE Healthcare). The results are expressed as the ratio of the relative expression to the untreated control.

### Measurements of Prostaglandin E_2_ and Cytokines Release

To explore the anti-inflammatory properties of recombinant proteins, we tested them under an inflammatory condition using IL-1β as inflammatory stimulus. Myoblast cells were seeded in six-well cell culture plates (Corning), and culture conditions and treatment with the recombinant proteins followed the protocol for inflammatory condition described above (Figure 1C). At the end of these treatments, cell-free supernatants were collected and kept at −80°C until evaluation of levels of PGE_2_ and cytokines. An enzyme-linked immunosorbent assay (ELISA) was performed to determine the concentration of prostaglandin E_2_ (PGE_2_) (Cayman Chemical). According to manufacturer’s instructions, samples were incubated overnight with the specific antibodies and conjugates. After the substrate solution was added, the plates were read at 405 nm in a spectrophotometer (SpectraMax). Culture supernatant levels of prostaglandins were interpolated from the standard curves, using a four-parameter logistic curve fitting.

Concentrations of the cytokines IL-2, IL-4, IL-6, IFN-γ, TNF, IL-17A, and IL-10 were measured using a cytometric bead array for mouse Th1/Th2/Th17 cytokines kit (BD Biosciences, San Jose, CA, United States). This multiplexed, beads-based immunoassay was performed according to manufacturer instructions to detect the cytokines. Briefly, capture beads were mixed and added to standards and samples, and then the phycoerythrin-detection reagent was added and incubated for 2 h at room temperature in darkness. Washing was performed by centrifugation at 200 *g* for 5 min and the pellets were suspended in the wash buffer. Then, standards and samples were acquired in a flow cytometer (FACS Calibur, BD Biosciences). Only relevant concentrations of IL-6 (pg/ml) were interpolated from the standard curves, using a five-parameter logistic curve fitting with the FCAP Array v3 software (BD Biosciences).

### Statistical Analysis

All experiments were performed in at least three independent times, some of them in technical duplicates. Data are expressed as the mean ± standard error of the mean (SEM). Statistical significance was determined using either one-way analysis of variance (ANOVA) tests with Dunnett’s post-test, or an unpaired t-test for direct comparisons. In addition, a two-way ANOVA with Dunnett’s post-test was used to analyse the myogenic regulatory factor expression in both the nucleus and the cytoplasm using GraphPad Prism 8.0.2 (GraphPad Software, La Joya, CA, United States).

## Results

### rLosac and rLopap Up-Regulate Myoblast Cell Proliferation

**FIGURE 2:**
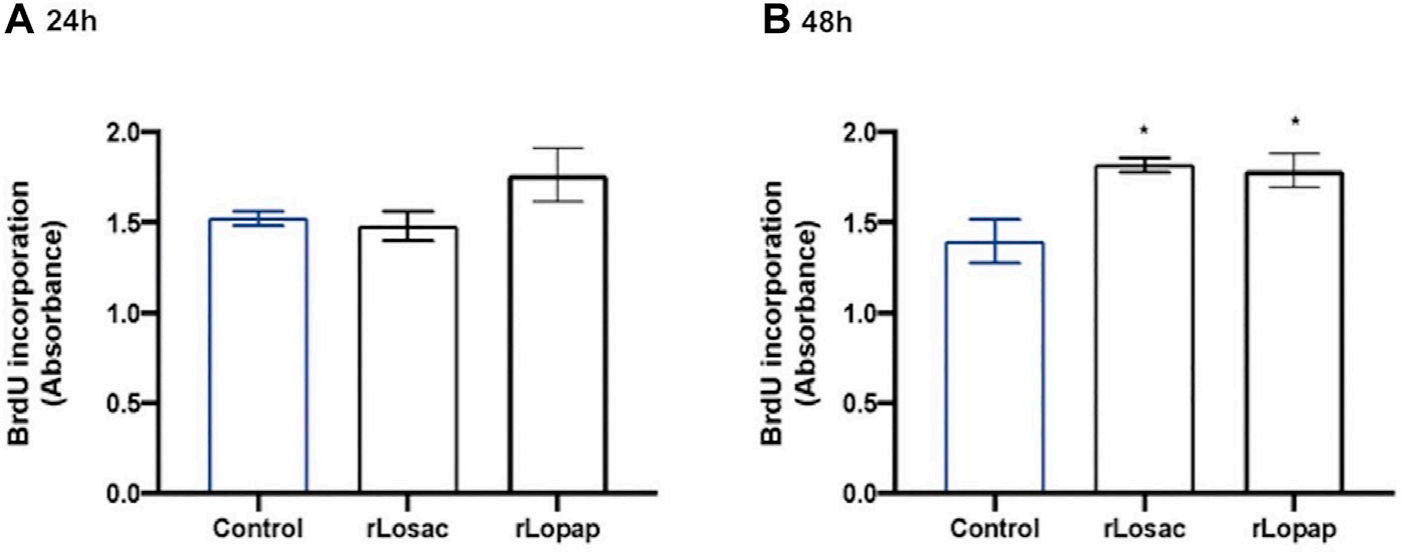
Recombinant proteins increased cell proliferation. C2C12 cells were treated with recombinant proteins in DMEM-1% ITS for 24 or 48 h, and then cell proliferation was assessed by incorporation of BrdU. Bar charts represent effects of control, rLosac and rLopap on BrdU incorporation at **(A)** 24 h and **(B)** 48 h. Data are shown as mean ± SEM (*p < 0.05 vs. control from one-way ANOVA and Dunnett’s post-test; *n* = 3 with technical duplicates).

Once activated, the satellite cells undergo active proliferation as the first step of the sequential processes that achieve muscle fibre repair or the formation of new myofibres (Schmidt et al., 2019; Forcina et al., 2020). Thus, we evaluated the effect of rLopap and rLosac in myoblast cell proliferation, assessed by 5-bromo-2′-deoxyuridine (BrdU) incorporation. We found that neither rLosac nor rLopap treatment modified myoblast cell proliferation when compared with control cells (treated only with culture medium) at 24 h (Figure 2A). However, at 48 h after treatment, both recombinant proteins significantly increased C2C12 proliferation (*p* < 0.05) when compared to control cells incubated with culture medium only (Figure 2B).

### rLosac and rLopap Modulates the Activity of Myogenic Regulatory Factors in Both Proliferated and Committed Cells

The MRFs modulate the stimulation, proliferation and differentiation of myogenic cells in a coordinated way (Singh and Dilworth, 2013; Chen and Shan, 2019; Schmidt et al., 2019), and cytoplasmic-nuclear trafficking of these factors is involved in that modulation (Brennan et al., 1991; Lingbeck et al., 2005; Lingbeck et al., 2008; Ferri et al., 2009; Crist et al., 2012). To better understand the stimulatory effects of rLosac and rLopap on the proliferation of myoblasts, we next evaluated the expression of the MRFs Pax7, Myf5, MyoD and myogenin upon treatment with the recombinant proteins, using high-content screening analysis (HCS). The differentiation protocol performed as illustrated in Figure 1B, showed mononuclear myocytes, which are typical cells of the initial phase of differentiation and are committed to fuse with each other to initiate the formation of myotubes. This initial phase is also named as committed stage of myoblast differentiation (Zammit et al., 2006; Carlson, 2021).

**FIGURE 3:**
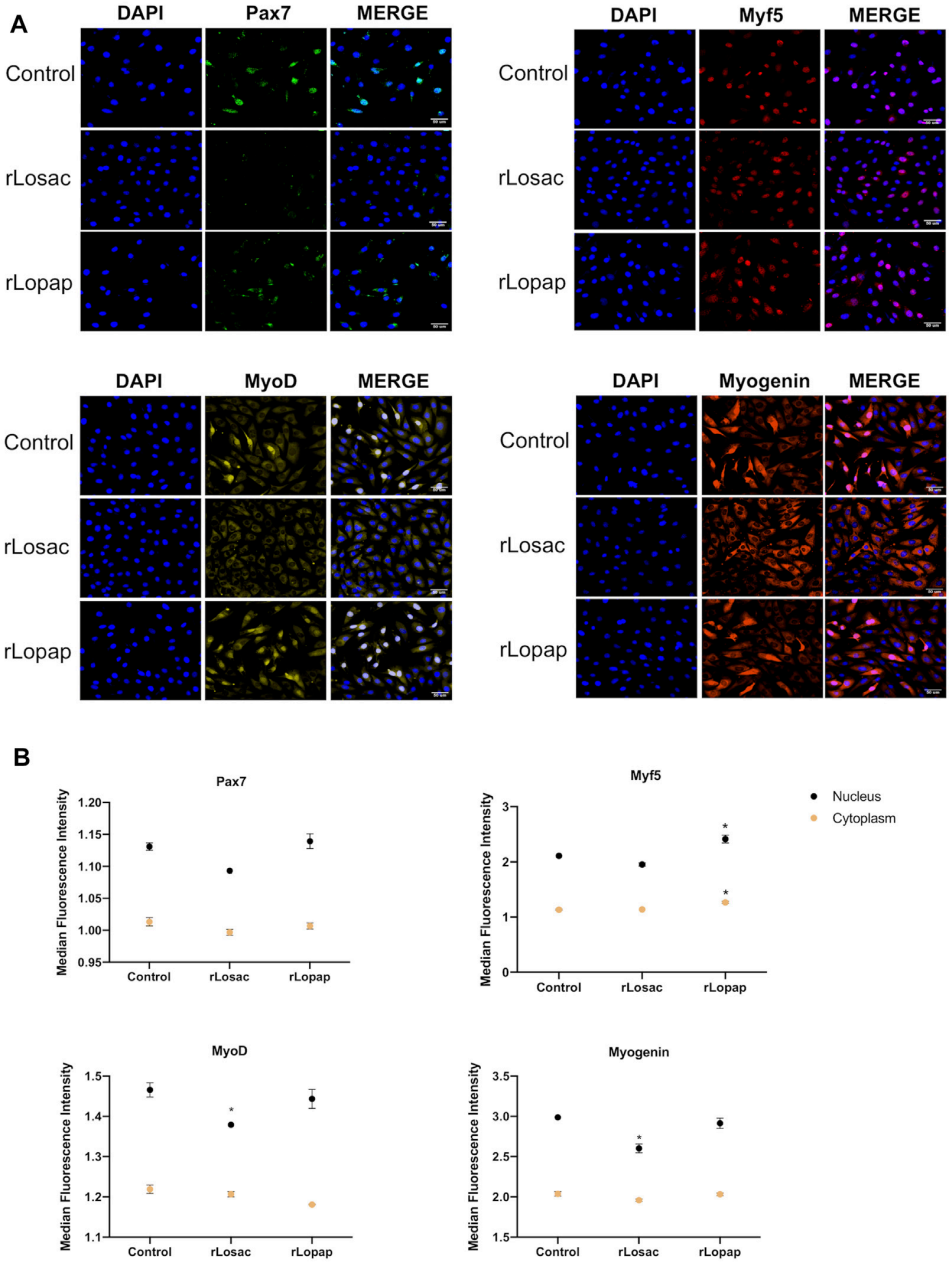
rLosac and rLopap modified the expression of myogenic regulatory factors (MRFs) at the proliferative stage. C2C12 cells were treated with recombinant proteins in DMEM-1% ITS for 24 h at the proliferative stage, and then MRF expression was assessed by high content screening (HCS). **(A)** Representative images of MRFs expression obtained by HCS (scale bar: 50 μm). **(B)** The average intensity in the nucleus (black) and cytoplasm (yellow) of every factor at basal (control) and treated conditions. Data are shown as mean ± SEM (*p < 0.05 vs. control from Two-way ANOVA and Dunette’s post test; *n* = 3 with technical duplicates).

**FIGURE 4:**
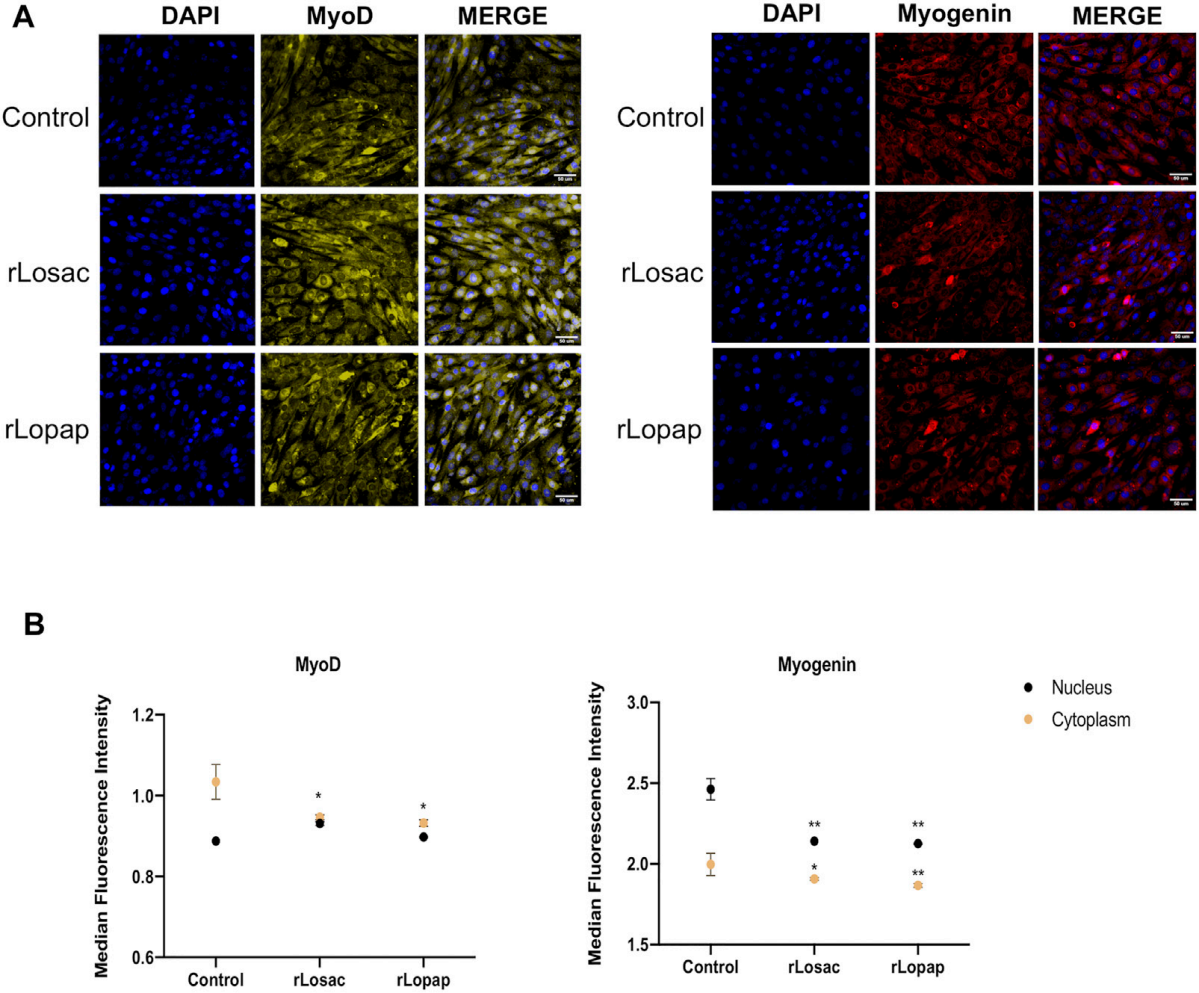
rLosac and rLopap modified the expression of MRFs at the commitment stage. Once the C2C12 cells reached the commitment stage were treated with recombinant proteins in DMEM-2% HS for 24 h, and then MFRs expression was assessed by HCS. **(A)** Representative images of MRFs expression obtained by HCS (scale bar: 50 μm). **(B)** The average intensity in the nucleus (black) and cytoplasm (yellow) of every factor at basal (control) and treated conditions. Data are shown as mean ± SEM (*p < 0.05 and **p < 0.01 vs. control from Two-way ANOVA and Dunnett’s post test; *n* = 3 with technical duplicates).

The representative images from HCS show that at the proliferative stage, Pax7, Myf5, MyoD and myogenin (Figure 3A), or MyoD and myogenin at the committed stage of myoblast differentiation (Figure 4A), are found at nuclear and cytoplasm sites indicating a dynamic state of their activation. At the proliferative stage, the quantification of fluorescence intensity showed that rLosac significantly (*p* < 0.05) decreased the nucleus expression of Pax7, MyoD and myogenin in comparison with control cells incubated with culture medium only (Figure 3B). Under the same experimental conditions, rLopap also stimulated the Myf5 activity by significantly increasing (*p* < 0.05) its translocation into the nucleus and its cytoplasmic expression. At the initial phase of cell differentiation, both recombinant proteins decreased cytoplasmic MyoD expression (*p* < 0.05) when compared with untreated control cells. Moreover, both rLosac and rLosac down-regulated the nuclear translocation and cytoplasmic expression of myogenin (*p* < 0.05), in comparison with untreated control cells (Figure 4B). Collectively, these results show the ability of recombinant Losac and Lopap to modulate key MRFs responsible for regulating the transition from the proliferated to committed stages of myoblasts.

### Cyclooxygenase and the PGE_2_ Pathway are Modulated by rLosac and rLopap on C2C12 in the Proliferative Stage

**FIGURE 5:**
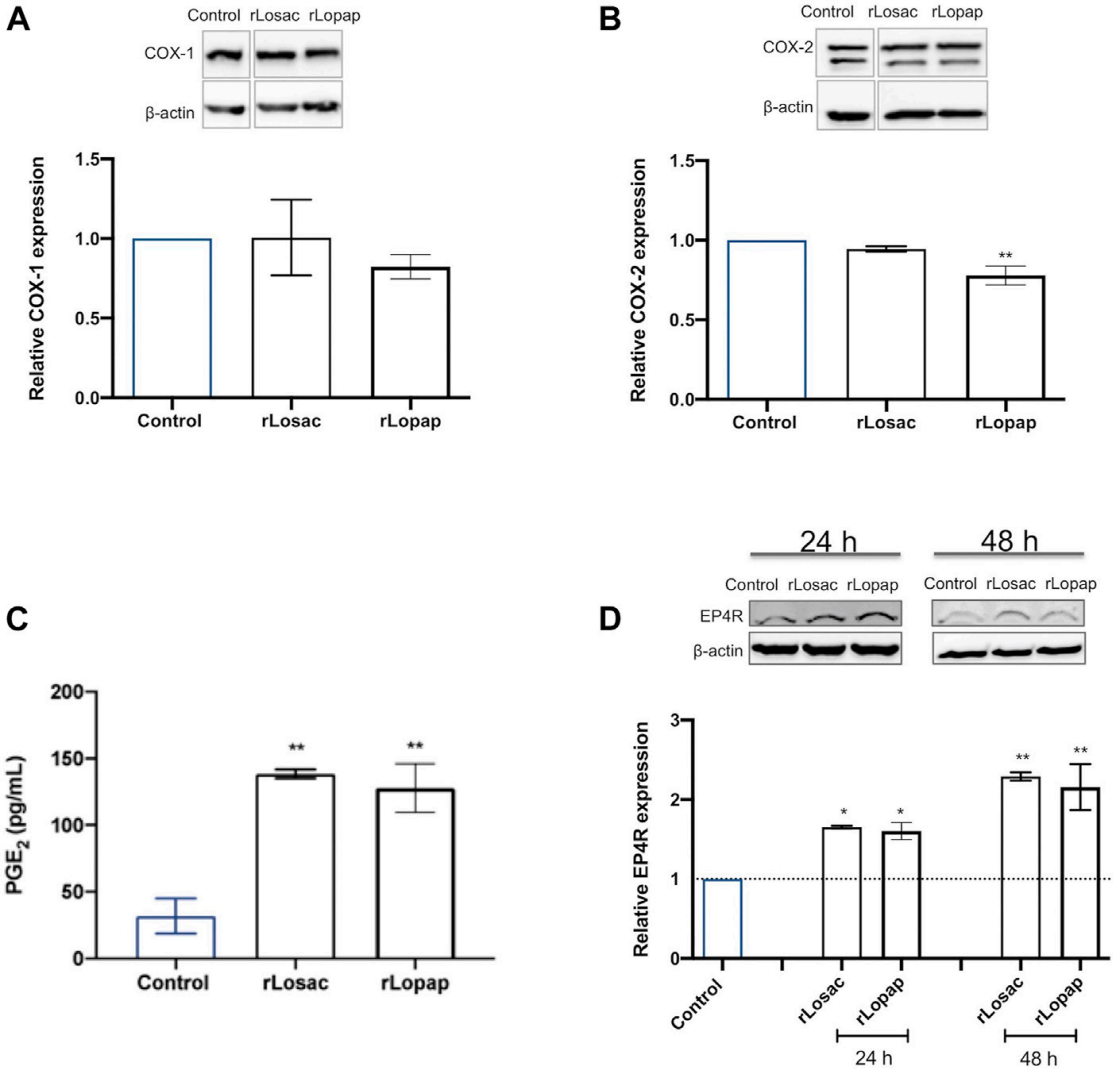
Recombinant Losac and Lopap affect the cyclooxygenase (COX) pathway. C2C12 cells were treated with rLosac or rLopap in DMEM-1% ITS for 24 h at the proliferative stage, and then Elisa and Western blotting respectively assessed prostaglandin E2 (PGE_2_) release, and COX pathway protein expression. Bar charts represent effects of control, and recombinant proteins on **(A)** COX-1 and **(B)** COX-2 protein expression, **(C)** production of PGE_2_ and **(D)** expression of EP4 receptor. Data are shown as mean ± SEM (*p < 0.05 and **p < 0.01 vs. control from one-way ANOVA and Dunnett’s post-test; *n* = 3).

The role of the COX pathway in skeletal muscle regeneration is well established, and mediators derived from this pathway are involved in modulating the proliferation and fusion steps of regeneration (Bondesen et al., 2004; Otis et al., 2005; Shen et al., 2006; Novak et al., 2009). Western blotting analysis showed that both rLosac and rLopap did not alter the protein expression of COX-1, in comparison with control cells (Figure 5A). Treatment of C2C12 cells with rLopap, but not rLosac, significantly reduced COX-2 protein expression (Figure 5B) when compared with untreated cells.

Next, PGE_2_ release was evaluated in cells treated with the recombinant proteins. As shown in Figure 5C, both rLosac and rLopap induced a significant increase in PGE_2_ release (128.01 ± 18.25 pg/ml and 138.40 ± 3.30 pg/ml respectively), in comparison with untreated cells (31.94 ± 13.11 pg/ml). Moreover, since the myogenic effects of PGE_2_ have been shown to be modulated by the EP4 receptor (EP4R) subtype (Mo et al., 2015; Ho et al., 2017), the effect of rLosac and rLopap on the protein expression of EP4R was evaluated. Results showed that treating C2C12 cells with either rLosac or rLopap caused a significant increase (*p* < 0.05) in EP4R protein expression after 24 and 48 h when compared to untreated control cells (Figure 5D).

### rLosac and rLopap Down-Modulate the IL-1β-Induced COX-1 Pathway and IL-6 Release

**FIGURE 6:**
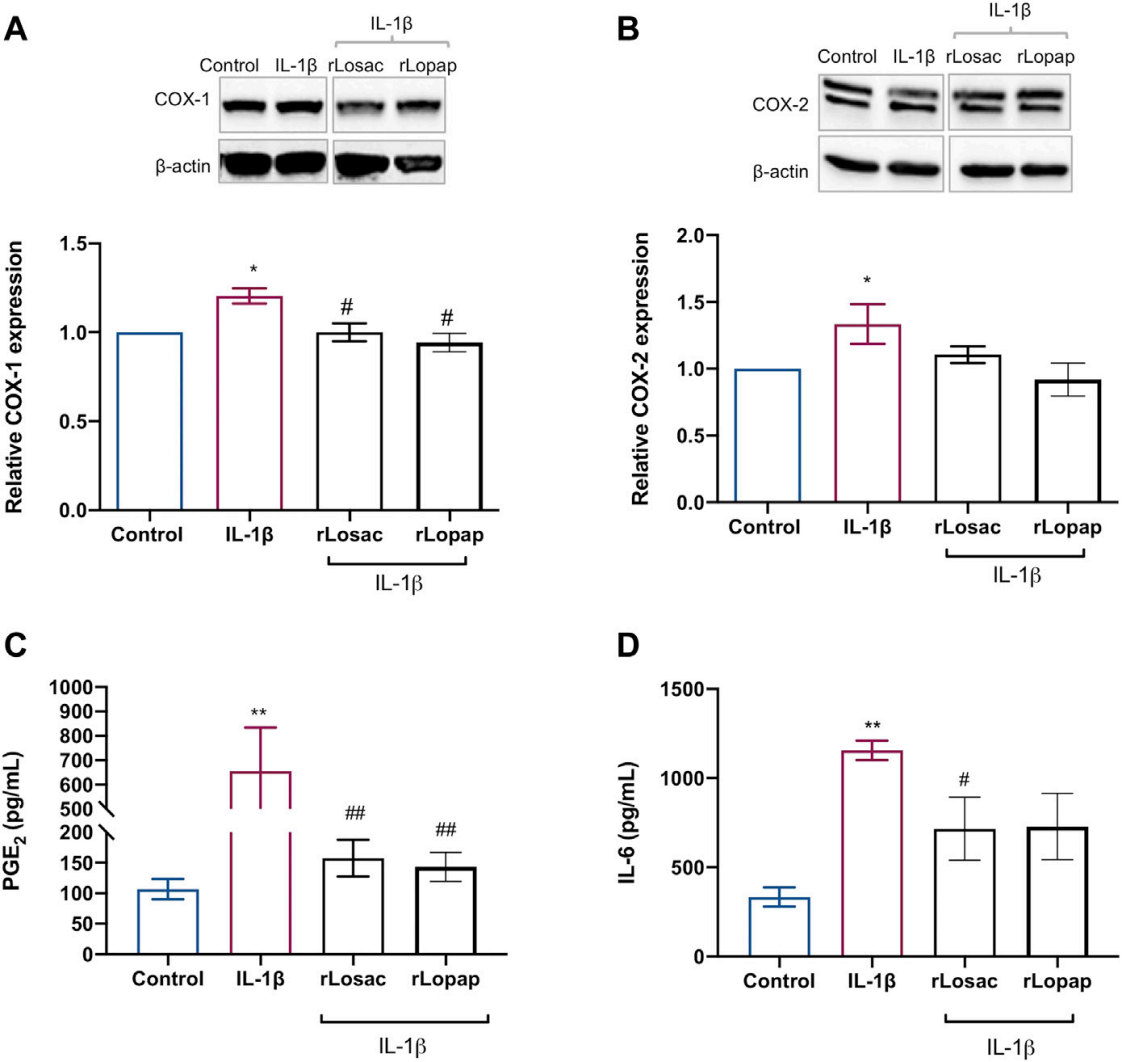
Recombinant Losac and Lopap have potential anti-inflammatory activity. C2C12 cells were treated with rLosac or rLopap in DMEM-1% ITS for 24 h for 24 h at the proliferative stage, and then PGE_2_ release, COXs proteins expression and interleukin 6 (IL-6) production were assessed by Elisa, Western blotting and flow cytometry respectively. Bar charts represent effects of control and recombinant proteins on the IL-1β-induced **(A)** COX-1 and **(B)** COX-2 protein expression, **(C)** PGE_2_ production and **(D)** IL-6 production. Data are shown as mean ± SEM (*p < 0.05 and **p < 0.01 vs. control; #*p* < 0.05 and ##*p* < 0.01 vs. IL-1β from one-way ANOVA with Dunnett’s post-test and unpaired t-test respectively; *n* = 3).

We evaluated the effect of the recombinant proteins on the IL-1β-induced release of PGE_2_ and COXs protein expression to assess the anti-inflammatory effect of rLosac and rLopap. The results, shown in Figure 6A, demonstrate that both recombinant proteins reduced (*p* < 0.05) the IL-1β-induced expression of COX-1, but the COX-2 expression was unaffected (Figure 6B) when compared with the respective control cells. Furthermore, as illustrated in Figure 6C, rLosac and rLopap significantly inhibited PGE_2_ production (157.6 ± 30.22 pg/ml and 143.1 ± 23.74 pg/ml, respectively) when compared to untreated cells stimulated with IL-1β (655.6 pg/ml ± 177.8). In addition, as demonstrated in Figure 6D, rLosac (716.2 ± 176.3 pg/ml) and rLopap (728.6 ± 185.7 pg/ml) markedly reduced the release of IL-6 in comparison with untreated cells stimulated with IL-1β (1156 ± 54.6 pg/ml).

## Discussion

Skeletal muscle has the inherent ability to recover after injury, through a highly synchronized process that depends on the ability of satellite cells to proliferate and differentiate. This process is dependent on molecular and transcriptional processes regulated by myogenic transcription factors (Schmidt et al., 2019). The transcription factor Pax7 is expressed by quiescent satellite cells and induces the expression of genes responsible for proliferation and commitment to the myogenic lineage in the initial regeneration process. These genes are activated downstream by the myogenic regulatory factors Myf5 and MyoD. Finally, the progression from commitment to terminal differentiation stages is evidenced by the activation of late markers, such as myogenin (Zammit et al., 2006; Ferri et al., 2009; Schmidt et al., 2019; Forcina et al., 2020).

By exploring the potential myogenic effects of rLosac and rLopap we found that both recombinant proteins up-regulated the proliferation of myoblast cells, a process that corresponds to the initial phase of skeletal muscle regeneration. To the best of our knowledge, this is the first report that rLosac and rLopap have the ability to stimulate a key process of skeletal muscle regeneration. The stimulatory effect of rLosac on myoblast proliferation is in line with previous reports that this protein promotes proliferation of human endothelial cells (Bettencourt et al., 1997; Bettencourt et al., 1999; Alvarez Flores et al., 2006; Alvarez-Flores et al., 2011; Bosch et al., 2016; Sato et al., 2016; Alvarez-Flores et al., 2019). The protein was therefore indicated by these authors as a promising molecule for the development of new formulations for wound healing.

To trace the mechanisms by which rLopap and rLosac stimulate the proliferation of myogenic cells, we investigated the modulation of different myogenic transcription factors by these biomolecules. Our results showed that rLosac decreased Pax7, MyoD, and myogenin activity in the proliferative stage, suggesting that the simultaneous decrease in activity of these factors may lead to the cell’s proliferative status and may restrain the differentiation process. This repressive effect was described in some satellite cell sub-populations, which downregulated MyoD activity once activated at the beginning of the regeneration process (Singh and Dilworth, 2013; Schmidt et al., 2019). In contrast, the increased Myf5 activity induced by rLopap in the proliferative stage is evidence of the distinctive molecular mechanism by which rLopap stimulates C2C12 cell proliferation, leading to myogenesis. Regarding the effects of the recombinant proteins in cells at the committed stage, we found that myogenin was expressed at higher levels than MyoD. This was expected, since myogenin initiates the terminal differentiation of myogenic progenitor cells accompanied by downregulation of MyoD (Chen and Shan, 2019; Schmidt et al., 2019). Indeed, both rLopap and rLosac decreased MyoD and myogenin activity, thus suggesting they act on proliferation rather than differentiation.

Although the structural derterminants were not investigated in the present study, the proliferative effects of rLosac and rLopap are probably related to their chemical structures. Hemolin is a protein involved in immunity, and it has been demonstrated that the hemolin gene has structural similarities with genes coding for cell-adhesion molecules (Lindström-Dinnetz et al., 1995). On the other hand, a hemolin derived from *Hyalophora cecropia* induced haemocyte aggregation–a mechanism observed during infection, wound or trauma–and was also expressed in developing tissues, oocytes and follicle cells (Bettencourt et al., 1997; Bettencourt et al., 1999), which suggest its participation in tissue and embryonic development. In addition, molecular docking studies revealed that the hemolin sequence from *Bombyx mori* has the highest affinity for the toll-like receptors (TLR) 3 and 4. A molecular dynamics simulation of the receptor-ligand complex confirmed these docking results and showed that hemolin-TLR4 has the stronger interaction (Aathmanathan et al., 2018). Interestingly, increased expression of TLR4 has been demonstrated in inflammatory myopathic tissues (Tournadre et al., 2010), and its constitutive expression has been described in C2C12 myoblasts and myotubes (Boyd et al., 2006; Cho et al., 2016). The activation of TLR4—among other TLRs–in cardiomyocytes results in the expression of IL-6 and other chemokynes *via* Nuclear factor kappa B (NF-κB) transcriptional activity (Boyd et al., 2006). In addition, activation of NF-κB up-regulates the expression of COX-2, as demonstrated in a mouse model of arthritis and rat macrophages (Khan and Khan, 2018; Liu et al., 2020). Thus, it may be suggested that an interaction between rLosac and the TLR4/NF-κB inflammatory pathway may play a role in the regulation of key mediators of myoblast function. Regarding the lipocalins, several have been reported to be involved in the mediation of cell regulation. In addition, synthetic peptides based on lipocalin motif 2, found in Lopap’s primary sequence, showed cytoprotective and antiapoptotic activity and increased the extracellular matrix proteins in both *in vitro* and *in vivo* approaches (Chudzinski-Tavassi et al., 2010; Carrijo-Carvalho et al., 2012). This suggests that motif 2 in Lopap may be involved in the stimulatory activity, demonstrated in our study, of this recombinant protein on the proliferation of C2C12 myoblasts. Furthermore, the existence of a lipocalin-type prostaglandin D synthase (PGDS) has previously been reported. This synthase differs in tissue distribution from haematopoietic-type PGDS, and its protective role was recently demonstrated in a KO mouse model of osteoarthritis (Najar et al., 2020).

It is known that prostaglandins derived from COXs pathway are involved in the modulation of the proliferation and fusion steps of muscle regeneration (Otis et al., 2005; Shen et al., 2006; Bondesen et al., 2007; Novak et al., 2009). Prostaglandins such as PGE_2_ and PGD_2_ exert various modulatory effects in the initial phases of muscular repair after injury (Veliça et al., 2010; Ho et al., 2017). In this regard, our findings demonstrating that rLopap and rLosac induced a prominent release of PGE_2_ from C2C12 cells in the proliferation stage suggest the participation of this mediator in the effects of these proteins on the proliferation of myoblasts. Since these recombinant proteins did not change the expression of COX-1 and only rLosac down-regulated COX-2 protein expression, an increase of COX-1 activity may be involved in the increased production of PGE_2_ seen upon treatment with either rLopap or rLosac.

In agreement with these results, Fritzen et al. (Fritzen et al., 2005), showed that Lopap stimulates the production of prostacyclin (PGI_2_) by the human endothelial cells HUVEC. Therefore, it is possible to suggest that the influence of products from the cyclooxygenase pathway on cell proliferation is modulated by rLosac in a tissue-specific manner. In line with these data, some studies have demonstrated that incubation of myogenic cells with prostaglandins, such as PGE_2_ and PGJ_2_, up-regulates the proliferation of myoblast cells (Duchesne et al., 2011; Mo et al., 2015). There are reports that PGE_2_ leads to myogenic events, such as the stimulation of myoblast proliferation, by mechanisms dependent on the activation of both COX-1 and COX-2 pathways, as demonstrated by a pharmacological approach with non-selective COX-1 and COX-2 inhibitors (Bondesen et al., 2004; Mendias et al., 2004; Otis et al., 2005; Silva et al., 2021). In addition, the crucial role of EP4 receptors in the PGE_2_-mediated activation of myoblast proliferation by mechanisms dependent on activation of cAMP-responsive element binding protein (CREB) pathway has been recently reported (Mo et al., 2015). In this context, our data showing that both rLosac and rLopap up-regulated the protein expression of EP4R in C2C12 in the proliferative phase, in a sustained manner, lead us to hypothesize that both recombinant proteins trigger muscle proliferative mechanisms *via* engagement of EP4 receptor by PGE_2_.

It is well known that during autoimmune diseases, such as rheumatoid arthritis and osteoarthritis, the skeletal muscle tissue is influenced by pro-inflammatory mediators released into its surroundings (Roubenoff, 2009; Costamagna et al., 2015; Huffman et al., 2017; Shorter et al., 2019). These can increase the production of other inflammatory mediators by the tissue itself, affecting the quality of muscle regeneration (Londhe and Guttridge, 2015). In this regard, the ability of the crude bristle extract of *Lonomia obliqua* to modulate inflammatory markers was recently demonstrated in a human macrophage model (Oliveira et al., 2021). As Lopap has previously been shown not to exert pro-inflammatory activity (Fritzen et al., 2005; Waismam et al., 2009) and, as with Losac, Lopap-derived peptides provide cytoprotective activity (Chudzinski-Tavassi et al., 2010; Carrijo-Carvalho et al., 2012; Wlian et al., 2014; Alvarez-Flores et al., 2021) and improve wound healing (Sato et al., 2016), we investigated the effect of these recombinant proteins in myoblast cells upon inflammatory stimulus by IL-1β. Interestingly, we found that both proteins exerted anti-inflammatory effects: they were able to inhibit the production of PGE_2_ and IL-6 induced by IL-1β. The inhibitory effect of both recombinant proteins on PGE_2_ production occurred by a mechanism involving down-regulation of the expression of the COX-1 isoform. Besides its important myogenic activity, PGE_2_ is a major pro-inflammatory mediator, associated with the potentiation of pain, vasodilation and oedema. IL-6, in turn, is a ubiquitously expressed cytokine that can have pro- and anti-inflammatory effects, depending on the concentration and local tissue environment (Sato et al., 2016). It is released during inflammatory processes and contributes not only to the immune response but also to the activation of metabolic and catabolic pathways, leading to a decrease in skeletal muscle mass (Belizário et al., 2016). The high expression of IL-6 causes muscle atrophy by cathepsin-dependent mechanisms, acting on the proteins of skeletal muscle and leading to tissue degradation (Belizário et al., 2016). Thus, the ability of rLopap and rLosac to decrease the production of IL-6 under inflammatory conditions implies an additional mechanism by which these proteins favour muscle proliferation.

In summary, our findings demonstrate that rLopap and rLosac have the ability to increase the proliferation of C2C12 myoblasts in culture at the proliferative stage. These recombinant proteins decrease Pax7, MyoD and myogenin activity, which may be the molecular mechanism involved in the stimulatory effect on myoblast proliferation. In addition, rLopap and rLosac induced the release of PGE_2_ but did not affect the expression of the inducible isoform COX-2, thus implying the involvement of COX-1 activity in the production of PGE_2_. Furthermore, the recombinant proteins markedly reduced the production of PGE_2_ and IL-6 upon inflammatory stimulus by IL-1β, evidencing their anti-inflammatory effect. These data together indicate that rLopap and rLosac can have a beneficial and protective action on skeletal muscle tissue, thus favouring skeletal muscle regeneration after injury.

## Data Availability

The raw data supporting the conclusions of this article will be made available by the authors, without undue reservation.
